# Rosetta Broker for membrane protein structure prediction: concentrative nucleoside transporter 3 and corticotropin-releasing factor receptor 1 test cases

**DOI:** 10.1186/s12900-017-0078-8

**Published:** 2017-08-03

**Authors:** Dorota Latek

**Affiliations:** Faculty of Chemistry, University of Warsaw, Pasteur St. 1, 02-093 Warsaw, Poland

**Keywords:** Membrane proteins, Solute carrier transporters, SLC28, Concentrative nucleoside transporter 3, Single nucleotide polymorphisms, G protein-coupled receptors, Corticotropin-releasing factor receptor 1, Rosetta Broker, GPCRM, NAMD

## Abstract

**Background:**

Membrane proteins are difficult targets for structure prediction due to the limited structural data deposited in Protein Data Bank. Most computational methods for membrane protein structure prediction are based on the comparative modeling. There are only few de novo methods targeting that distinct protein family. In this work an example of such de novo method was used to structurally and functionally characterize two representatives of distinct membrane proteins families of solute carrier transporters and G protein-coupled receptors. The well-known Rosetta program and one of its protocols named Broker was used in two test cases. The first case was de novo structure prediction of three N-terminal transmembrane helices of the human concentrative nucleoside transporter 3 (hCNT3) homotrimer belonging to the solute carrier 28 family of transporters (SLC28). The second case concerned the large scale refinement of transmembrane helices of a homology model of the corticotropin-releasing factor receptor 1 (CRFR1) belonging to the G protein-coupled receptors family.

**Results:**

The inward-facing model of the hCNT3 homotrimer was used to propose the functional impact of its single nucleotide polymorphisms. Additionally, the 100 ns molecular dynamics simulation of the unliganded hCNT3 model confirmed its validity and revealed mobility of the selected binding site and homotrimer interface residues. The large scale refinement of transmembrane helices of the CRFR1 homology model resulted in the significant improvement of its accuracy with respect to the crystal structure of CRFR1, especially in the binding site area. Consequently, the antagonist CP-376395 could be docked with Autodock VINA to the CRFR1 model without any steric clashes.

**Conclusions:**

The presented work demonstrated that Rosetta Broker can be a versatile tool for solving various issues referring to protein biology. Two distinct examples of de novo membrane protein structure prediction presented here provided important insights into three major areas of protein biology. Namely, the dynamics of the inward-facing hCNT3 homotrimer system, the structural changes of the CRFR1 receptor upon the antagonist binding and finally, the role of single nucleotide polymorphisms in both, hCNT3 and CRFR1 proteins, were investigated.

**Electronic supplementary material:**

The online version of this article (doi:10.1186/s12900-017-0078-8) contains supplementary material, which is available to authorized users.

## Background

Structure prediction of small (up to 150 amino acids) globular proteins has improved so much that it has become nearly as accurate as low resolution experimental methods [1]. However, there is still a serious bottleneck in membrane protein structure prediction. The number of membrane protein structures deposited in Protein Data Bank (PDB) is much smaller than that for globular proteins. As a consequence, PDB provides relatively weak statistics for membrane proteins. There are two families of membrane proteins which still lack adequate characterization though they represent important drug targets. The first family is a well-known family of G protein-coupled receptors (GPCRs) which share a common structural motif of seven transmembrane helices. The second one is less known and more structurally diverse family of solute carrier transporters (SLCs).

For many years G protein-coupled receptors have been drug targets for many diseases including neurological, cardiovascular, endocrinological disorders. Structure prediction of GPCRs using template structures from the same GPCR subfamily, e.g., rhodopsin-like, frizzled or secretin GPCRs, proved to be accurate enough for drug design in many cases (see, e.g., results of GPCR Dock competitions [1–3]). Furthermore, there are many tools and web services for automatic structure prediction of GPCRs, e.g., GPCRM [4, 5], GPCR-Tasser [6], GPCRMod-sim [7], GOMODO [8], etc. In general, the GPCR homology modeling includes the following steps: selection of a template structure providing a proper deformation of transmembrane helices (kinks, bulges, etc.), alignment generation and finally loop refinement which greatly affects ligand binding [5]. Despite the recent progress in the GPCR modeling, reliable structure prediction of GPCRs based on distant homology (the SMO receptor case in GPCR Dock 2013 [1]) or prediction of the opposite activation state (the 5-HT_2B_ receptor case in GPCR Dock 2013), is still out of reach for the majority of researchers.

Structures of 52 human families of SLCs consisting of 386 proteins are less known than GPCRs. SLCs are integral transmembrane proteins through which endogenous (i.a. ions, nucleotides, peptides) and exogenous substances (i.a. xenobiotics, drugs and their metabolites) are transported down or against the electrochemical gradient by coupling the transport with the flow of Na + or H+ [9]. SLCs are involved in drug absorption, distribution, metabolism and excretion (ADME). What is more, they can be drug targets themselves, e.g., in cancer and antibacterial pharmacotherapies [9]. SLCs influence the effectiveness of pharmacotherapy and the occurrence of drug side effects and drug-drug interactions [9, 10]. Recently, it was also proved that SLCs are important for pharmacogenomics studies [9–11]. For example, single nucleotide polymorphisms (SNPs) observed in the SLC47 family of transporters affect the pharmacotherapy of diabetes type II in the ethnical group of Latin Americans [12]. Depending on the localization of their expression SLCs can play different roles. For example, SLCs expressed in intestinal cells, hepatocytes and cells of the brain-blood barrier play an important role in nutrition absorption and protection against xenobiotics [9, 10]. On the other hand, SLCs localized in kidneys and liver cells are involved in excretion of drugs and their metabolites [9, 10].

All SLCs are alpha-helical membrane proteins but structure and sequence similarity among members of different SLC families is limited [9]. For example, multidrug and toxin extrusion protein 1 (MATE1) from SLC47 share only 19.0%, sodium-coupled neutral amino acid transporter 1 (SLC38) 15.9% and riboflavin transporter (SLC52) 13.6% sequence identity with hCNT3 from SLC28 (data obtained with Clustal-Omega [13]. On the other hand, TM-score [14] between the corresponding crystal structures of close homologs of vcCNT (PDB id: 3TIJ) and MATE1 (PDB id: 4HUK) is only 0.288 with only 213 aligned residues (out of total 459 residues of 4HUK) with heavy atom RMSD equal to 6.85 Å. What is more, SLCs are too large to be studied only by de novo methods [15, 16]. For that reason, recent theoretical studies [16–18] on SLCs involve only homology modeling with template structures from bacterial organisms sharing at least 20% sequence identity with targets or additional data from experiments [19, 20]. Another problem in the homology modeling of SLCs is a significant structural difference between their inward-facing occluded conformation and the outward-facing conformation. The change between inward and outward-facing conformation during the transport process through the cell membrane requires not only transmembrane helices deformation like in the case of the GPCR activation but also a large change in the transporter topology (see so-called inverted topology [21]). Forrest et al. proposed the first solution to this problem called the repeat swap technique [22], first applied to the LeuT transporter from the SLC6 family [23]. Recently, her group also calibrated that technique using known crystal structures of inward- and outward-facing conformations of the GltPh transporter and successfully used it for studying the elevator-like model of the vcCNT transport mechanism [22].

**Fig. 1:**
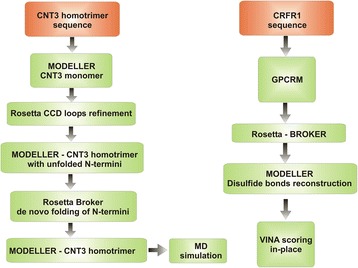
The algorithm description

Difficulties in structure prediction of GPCRs and SLCs experienced when there was no close homologous structure in PDB prompted the scientific community to search for new computational methods. A versatile example of such novel method is Broker (also known as Topology Broker) implemented as an extension of the well-known Rosetta program [24, 25]. In this manuscript, Broker together with GPCRM [4, 5] and MODELLER [26] (see Fig. 1) was used to model de novo transmembrane helices of the SLC transporter and to impose native-like deformations of transmembrane helices of the G protein-coupled receptor.

Two test cases were selected: the human CNT3 transporter in the inward-facing conformational state and the CRFR1 receptor in its inactive state. CNT3 together with CNT1 and CNT2 form the SLC28 family of concentrative nucleoside transporters. CNTs actively transport nucleosides or nucleoside-derived drugs, e.g., anticancer gemcitabine or antiviral ribavirin, by coupling their transport to the movement of Na + ions towards inside of the cell [9, 27]. The validity of the hCNT3 homotrimer model constructed in this work was assessed with the 100 ns molecular dynamics (MD) simulation with explicit membrane. The second case tested here was the corticotropin-releasing factor receptor type 1 (CRFR1). CRFR1 is a G protein-coupled receptor from the secretin-like GPCR family (the former class B of GPCR receptors) [28]. It mediates the stress response and is known as a molecular target in the treatment of depression and anxiety. The model of CRFR1 was compared to the crystal structure of CRFR1 (PDB id: 4K5Y) and used in a small molecule docking experiment. Finally, genetic variations associated with the presented protein structures were discussed. Namely, in both, hCNT3 and CRFR1 models several functionally important residues associated with single nucleotide polymorphisms (SNPs) were localized. A potential impact of SNPs on the functioning of hCNT3 and CRFR1 proteins was hypothesized.

## Methods

### CNT3 model building

**Fig. 2:**
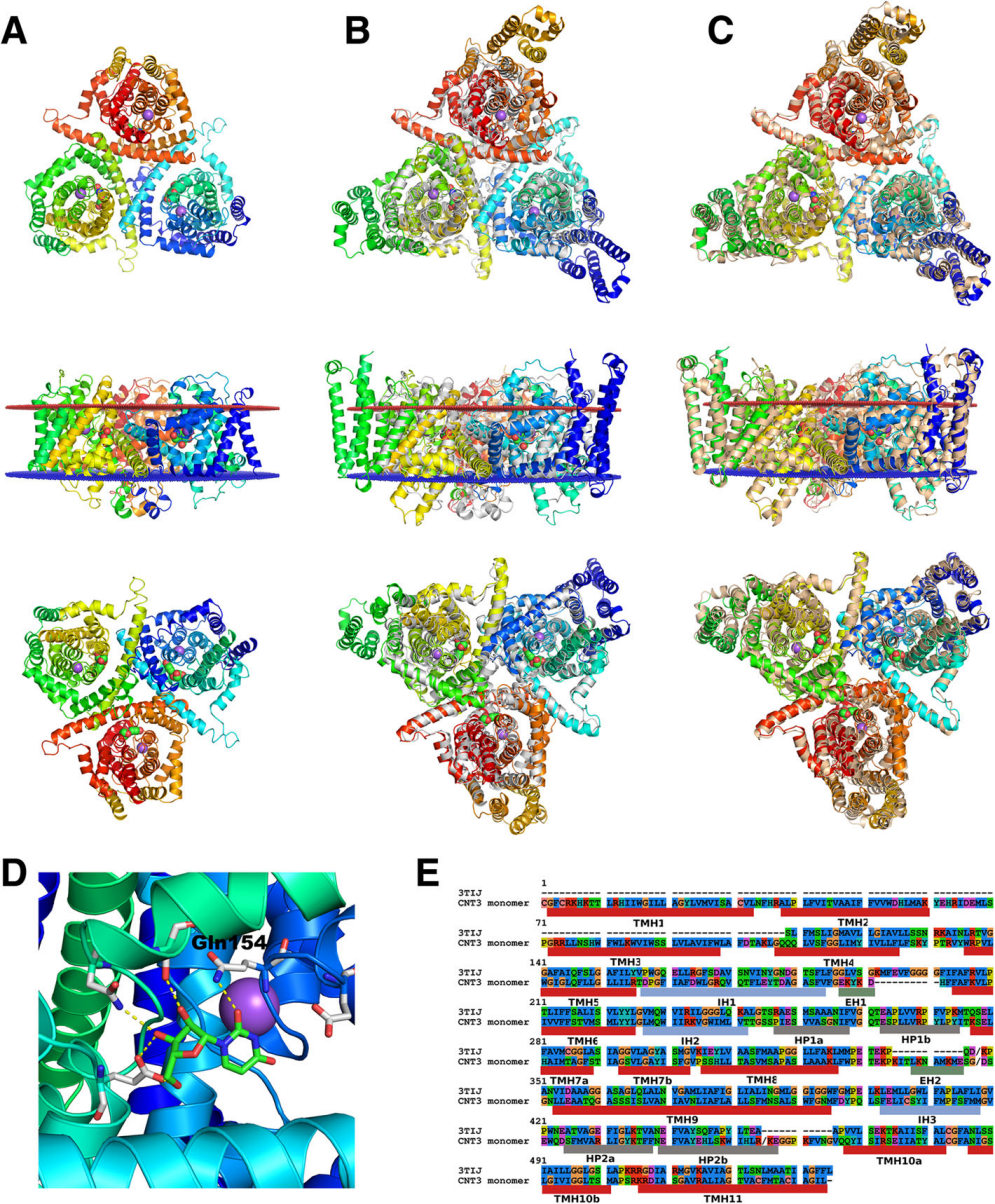
**a** The crystal structure of the vcCNT homotrimer (PDB id: 3TIJ) shown in the extracellular, membrane and intracellular view, respectively. **b** A homology model of the hCNT3 homotrimer superposed on the crystal structure of vcCNT (grey) shown in the extracellular, membrane and intracellular view, respectively. **c** A homology model of the hCNT3 homotrimer superposed on the low-energy structure obtained from the 1956 frame out of all 5000 frames of the 100 ns MD simulation, shown in the extracellular, membrane and intracellular view, respectively. **d** The binding site of the uridine molecule (shown in green) and the sodium ion (shown as a violet sphere) located inside the crystal structure of vcCNT. The polar contacts between uridine and the transporter were depicted with yellow dashed lines. The indicated Gln154 in vcCNT corresponds to Gln251 in the model of hCNT3. **e** The sequence alignment of the template sequence (vcCNT) and the target sequence (hCNT3). Transmembrane helices (TMHs) are shown in red, extracellular and short helices (EH) in green, amphipathic helices (IH) are shown in blue and finally helices outside the lipid bilayer (HP) are shown in grey

To build the hCNT3 model the standard automodel routine of MODELLER-9v11 [26] and the vcCNT template structure (PDB id: 3TIJ) were used. A small molecule ligand uridine, a sodium ion and two water molecules which were present in the binding site of the crystal structure of vcCNT (see Fig. 2d) were also added. Thus, the proper orientation of side chains inside the hCNT3 binding site was preserved during the model building procedure. To build the model of the hCNT3 monomer only the fragment of the full 691-residue long sequence of hCNT3 (Uniprot id: Q9HAS3) was used. Namely, the N and C-terminus which were predicted to be outside the membrane (see Uniprot) were cut out leaving the 522-residue hCNT3 sequence (see Fig. 2e) corresponding to the residue range 91 – 612 from the Q9HAS3 entry. The lowest energy model, according to the DOPE energy function, of the hCNT3 monomer out of 100 generated was selected and used in the subsequent loop refinement. The refinement of the hCNT3 monomer loops was performed in Rosetta3 using the cyclic coordinate descent algorithm (CCD) [29]. To preserve efficiency of sampling of conformational space loop refinement simulations were divided in three separate categories. The first one was dedicated to the loop refinement of the 185 - 194 sequence region, the second one to the 128 - 136, 234 - 237, 258 - 266 and 317 - 341 sequence regions and the third one to the 486 - 493 sequence region. In each category 1000 loop models were generated. All 1000 models generated in each loop category were subjected to the clustering analysis with the Rosetta cluster application. From each category 20 cluster representatives, each of which had the lowest total Rosetta score within its cluster, were selected. All the cluster representatives were combined with each other to generate 8000 (20 × 20 × 20) possible loops combinations. Each loop combination was used to build one model of the hCNT3 homotrimer using the vcCNT template structure (PDB id: 3TIJ) and the MODELLER procedure described above. Here, the 3-fold symmetry of the hCNT3 homotrimer was kept. The DOPE potential was used to select the best model of hCNT3 out of all 8000 generated. That 1566-residue long hCNT3 model (all three subunits: 3 × 522 residues) was cut to the 1350-residue long model by removing N-termini of the subunits B and C. That 1350-residue long model of hCNT3 was subjected to de novo folding of N-terminus of the subunit A with Rosetta Broker [25]. For the Broker simulation all standard settings for Rosetta3 were used (see Additional file 1: Table S1–S2). Namely, implicit membrane energy terms described in details in [30] and the fragment library (3- and 9-residue long fragments) obtained with Robetta (http://robetta.bakerlab.org/fragmentsubmit.jsp) were used. The consensus membrane topology predictor TOPCONS [31] and the hCNT3 Uniprot entry (id: Q9HAS3) were used to detect positions of three N-terminal transmembrane helices (TMHs) (see Fig. 2e). Additionally, the sequence profile-based lipophilicity prediction was performed and used in the Broker simulation. During the Broker simulation only the N-terminal 108-residue long fragment in the first subunit A with the predicted three TMHs was kept flexible. The rest of the homotrimer was kept as a rigid body. Nevertheless, various approaches were tested (data not shown) before the final modeling protocol was decided. Namely, longer N-terminal fragments, 198- and 247-residue long, including the 90- and 139-residue long membrane regions of hCNT3 were folded de novo without the rest of the hCNT3 homotrimer. Also, the short, 108-residue long N-termini only in the presence of the subunit A structure was folded. Yet, it turned out that the best option for the Broker simulation was folding of the short, 108-residue long N-termini of the subunit A with the presence of other subunits B and C forming the whole 1350-residue long hCNT3 homotrimer. 10,000 models were generated and clustered using the Rosetta3 cluster application. Top ten low-energy models from the most populated cluster of the hCNT3 models according to the Rosetta total score were selected and visually inspected. One selected model was used as a template to build the final hCNT3 homotrimer model with the described above MODELLER procedure. The N-terminal region with three TMHs predicted de novo was repeated in all three subunits to ensure the 3-fold symmetry of the homotrimer. A total number of 20 hCNT3 homotrimer models were generated and the lowest energy model according to DOPE was subjected to the further analysis and the MD simulation.

### Molecular dynamics simulation

**Fig. 3:**
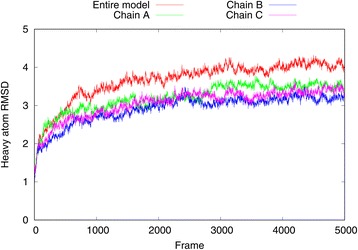
The heavy atom RMSD plot computed for all 5000 frames recorded during the 100 ns MD simulation. RMSD was computed for the entire hCNT3 homotrimer and its three subunits with respect to the first frame of the MD simulation

**Fig. 4:**
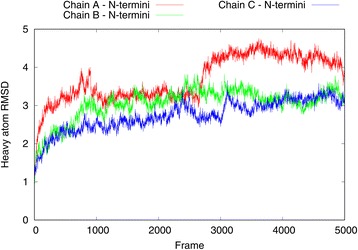
The heavy atom RMSD plot computed for all 5000 frames recorded during the 100 ns MD simulation. RMSD was computed for N-terminal regions including three transmembrane helices located in all three hCNT3 subunits with respect to the first frame of the MD simulation. At the end of the simulation all N-terminal regions are of 3.5 Å RMSD

**Fig. 5:**
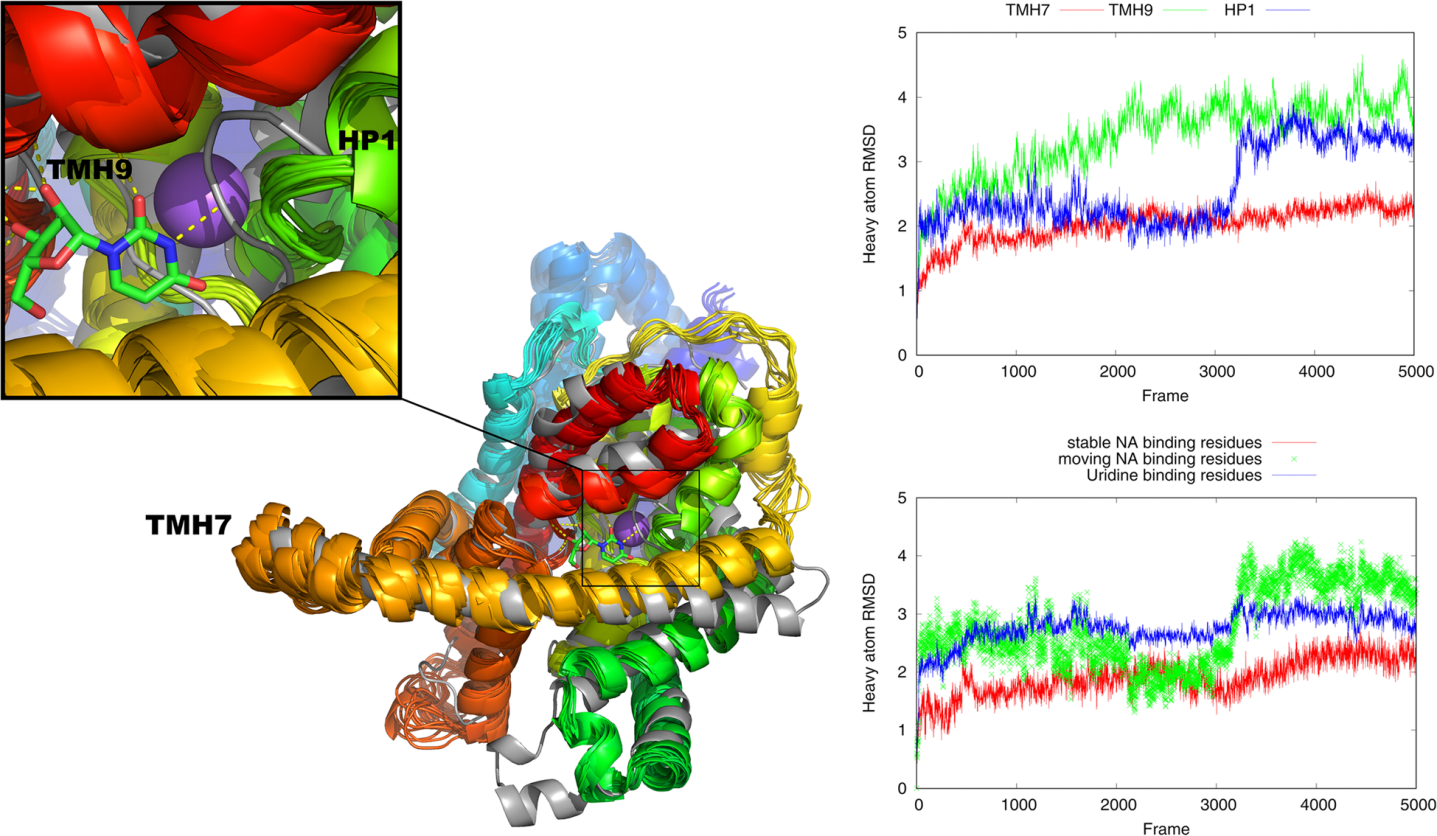
The left panel: an ensemble of the low energy conformations of the hCNT3 subunit A (grey) extracted from the last 40 ns of the 100 ns MD simulation trajectory superposed on the crystal structure of vcCNT (blue-to-red). The template structure of vcCNT includes uridine (shown in the sticks representation) and sodium ion (a violet sphere). Polar contacts between uridine and vcCNT were shown as yellow dashed lines. The enlarged binding site is shown on the top left. Three helices of hCNT3 were indicated: HP1, TMH7 and TMH9. The right panel: the heavy atom RMSD plot computed for the MD simulation trajectory with respect to the first frame. RMSD was computed for the selected helices (the right top plot) and the binding site residues (the right bottom plot)

**Fig. 6:**
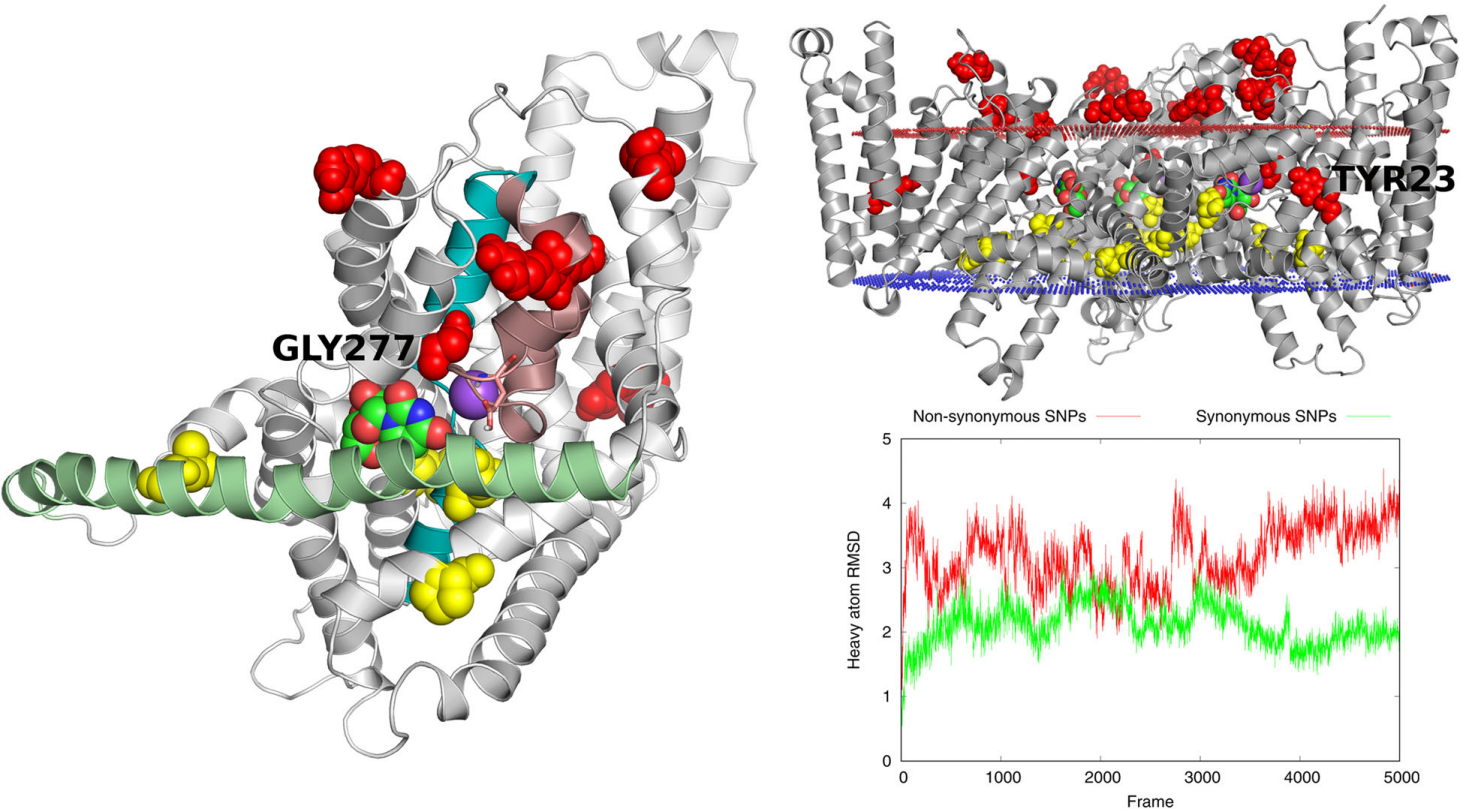
Characterization of SNPs in the hCNT3 homotrimer model. Residues to which non-synonymous mutations refer were shown as red balls, residues referring to synonymous mutations were shown as yellow balls. The left panel: a single hCNT3 subunit with uridine (green balls) and sodium ion (violet ball) which were transferred from the vcCNT template structure and placed in the same position and orientation. The right panel: the entire hCNT3 homotrimer model with the schematic lipid bilayer (red and blue dots). The right bottom panel: the heavy atoms RMSD plot computed for the MD simulation trajectory with respect to the first frame. RMSD was computed for two residues sets – referring to synonymous mutations and to non-synonymous mutations

The MD simulation was performed using the GPU-accelerated NAMD [32] software with the CHARMM27 [33] all-atom force field and periodic boundary conditions. Electrostatic interactions were computed using the particle-mesh Ewald method (PME) with a real space cutoff of 1.0 nm. The Lennard-Jones interactions were also cut off at 1.0 nm. The hCNT3 homotrimer model was inserted in a pre-equilibrated palmitoyloleoylphosphatidylcholine (POPC) membrane with VMD [34, 35]. The final lipid membrane was composed of 349 lipids. The system was solvated using the TIP3P water model (41,236 water molecules) and neutralized by adding 35 chloride counterions. Aspartic acid, arginine, glutamic acid, and lysine residues were used in their physiological protonation states. Neither uridine nor sodium ion molecules which were present in the vcCNT template structure were added to the system. The final system contained a total number of 195,438 atoms. The equilibration phase started with the 1 ns long melting of lipid tails while the rest of the system remained fixed. Then, after the steepest descent system minimization only protein coordinates were harmonically restrained and the 2 ns equilibration of the whole system was performed. Finally, the harmonic constraints were released and the further equilibration of the whole system lasted for 2 ns. The size of the final periodic box after the equilibration phase was 14.8 nm × 14.5 nm × 105 nm. The 100 ns production run was executed using a 2 fs time step with a snapshot of the system conformation and its energy saved every 20 ps and 10 ps, respectively. The pressure control was provided by using a modified Nosé-Hoover method in which Langevin dynamics is used to control fluctuations in the barostat. The thermostat was provided by Langevin dynamics with damping coefficient of 1/ps. The simulation was conducted at the conditions of 300 K and 1 atm. RMSD plots (see Figs. 3, 4, 5 and 6) describing the hCNT3 behavior during the MD simulation were prepared with VMD.

### CRFR1 model building

To build the CRFR1 model a standalone version of GPCRM described previously [5] was used. The human glucagon receptor (GCGR) structure (PDB id: 4L6R) [36] from the secretin-like branch of the GPCR family was selected as a template. To generate the CRFR1 model a PDB sequence was used (PDB id: 4K5Y, Uniprot entry: P34998, isoform 2 – CRF-R2). The isoform 2 differs from the canonical CRF-R1 sequence only in such way that a part of the sequence is missing. GPCRM generated 3000 models. Only one out of the ten best models proposed by GPCRM was selected for the next stage based on the RMSD criterion referring to the crystal CRFR1 structure (PDB id: 4K5Y). The membrane topology prediction for the Rosetta Broker input was extracted directly from the CRFR1 model. The Broker simulations were divided into 3 stages. In the first stage, only the N-terminal fragment of the transmembrane helix 1 (TMH1) was reconstructed (2000 models) and the lowest RMSD model was selected. In the next step, TMH2, TMH3, TMH4, TMH5, TMH7 were rebuilt (30,000 models) and again the lowest RMSD model with respect to the crystal structure of CRFR1 was selected. In the final step of the Broker simulation TMH6 was reconstructed to fit the native structure [28] of CRFR1 (20,000 models). As it was tested before [5] the best way to impose disulfide bonds in a GPCR model is to use MODELLER. For that reason, the last modeling stage was devoted to the MODELLER reconstruction of disulfide bonds which were slightly deformed during the Broker simulation (100 models). The lowest MODELLER objective function model was selected for the antagonist docking in Autodock VINA [37].

As it was mentioned above, the main selection criterion in all the CRFR1 modeling stages was RMSD with respect to the CRFR1 crystal structure (PDB id: 4K5Y). The reason for that was the main purpose of the current work. Namely, the current work was not focused on the assessment of the Rosetta Broker force field accuracy. The accuracy of knowledge-based force fields in the membrane protein structure prediction is an important topic [38] but outside the scope of this study. Here, only the best possible results which could be obtained with the current force field and the current sampling algorithm implemented in Broker were examined. That is why only the RMSD criterion was used and not the energy criterion for the CRFR1 models selection.

### Small molecule docking

The binding mode of the CRFR1 antagonist CP-376395 is well described in [28] and the current study was not focused on the antagonist docking itself. Instead, this work was focused on the assessment of the quality of the CRFR1 homology model in the binding site area and detection of possible atom clashes. For that reason, the CP-376395 molecule was placed exactly in the same position inside the CRFR1 homology model as in the crystal CRFR1 structure. What is more, only the local refinement of the binding site was performed with Autodock VINA [37] before computing the value of the empirical docking scoring function which estimated the free energy of the ligand binding. The free energy of the antagonist binding which reflected steric clashes between atoms [37] was provided for three cases. The first case was the crystal structure of the CRFR1 complex with the CP-376395 antagonist (PDB id: 4K5Y). The second case was the template-based CRFR1 model built by GPCRM with CP-376395 transferred from the crystal CRFR1 structure and placed exactly in the same position and orientation. The third case was the CRFR1 model built by GPCRM but refined with the Broker algorithm with CP-376395 transferred from the crystal CRFR1 structure (PDB id: 4K5Y). In the all three cases the standard Autodock VINA settings were used together with the local_only option and the 20Åx20Åx20Å searching space size.

### Single nucleotide polymorphisms

Single nucleotide polymorphisms (SNPs) for hCNT3 were downloaded from the UCSF Pharmacogenetics of Membrane Transporters (PMT) database (http://pharmacogenetics.ucsf.edu) (HGNC id: 16,484, HGNC symbol: SLC28A3) [39]. SNPs for the CRFR1 receptor were obtained from the National Institute of Health Short Genetic Variations database (dbSNP) [40] (id: 1394) and refer to the isoform 1 (CRF-R1). Nevertheless, sequence numbering for SNPs was adjusted to fit the isoform 2 sequence (CRF-R2) which was used to build the CRFR1 model and was included in the PDB entry for that receptor (PDB id: 4K5Y).

## Results and discussion

### CNT3 transporter

In 2012 the first crystal structure of the transporter from the SLC28 family was solved [9]. It was the structure of the bacterial vcCNT transporter isolated from Vibrio cholerae (PDB id: 3TIJ). In 2014 another crystal structure of vcCNT was released in PDB [27]. However, the current study had been started before releasing of the 2014 structure so the 2012 structure was used as a template. The vcCNT transporter is crucial for toxin excretion and plays an important role in antibiotic resistance. Most probably, CNTs change their conformations during the transport according to the elevator-like mechanism [41]. The PDB entry 3TIJ represents an inward-facing occluded conformation of the vcCNT transporter and thus represents a suitable template to build a model of the inward-facing conformation of the human CNT3 transporter (hCNT3). Human hCNT3 and bacterial vcCNT sequences share 39.46% sequence identity (according to the Clustal Omega web service [42]). Therefore, the homology modeling of hCNT3 using vcCNT as the template structure can be described as relatively easy [43, 44]. An important difference in the binding site area between hCNT3 and vcCNT is the presence of two cysteine residues Cys471 and Cys512 in hCNT3 which are so close to each other that they could form a disulfide bond. However, Slugoski et al. [45] showed that the cysteinless hCNT3 transporter still retained wild-type functional activity yet with the increased K50 dissociation constant for the sodium ion binding. What is more, CNT1 and CNT2 human homologs of CNT3 lack cysteines in that sequence region (data not shown). So, most probably that disulfide bridge is not present in the hCNT3 binding site as it was not preserved evolutionary. Therefore, the model of the hCNT3 homotrimer presented in this work was built with two non-bonded cysteines inside the binding site.

The vcCNT template structure did not correspond to the full human CNT3 sequence. There were three transmembrane helices in the N-terminal part of the hCNT3 sequence (see Fig. 2e) which were not present in vcCNT. In this work, those three TM helices were reconstructed de novo with Rosetta Broker [24, 25]. In principle, such de novo algorithm as Rosetta Broker is able to generate protein structures from families which are absent or poorly populated in PDB. That is especially important for the case of membrane protein family. The protein representation used in Broker and in other Rosetta protocols relies on internal coordinates (bond lengths, angles and torsions) which makes Rosetta a highly scalable algorithm [24]. Broker is also very efficient regarding the computational time and the conformational space sampling. That is due to a specific design of its programming architecture managing the interplay between variety of sampling strategies (so-called Movers) and the central broking mechanism [24]. The specific broking mechanism enables to simulate large protein systems consisting of many domains or monomers of various symmetry types (see the “fold-and-dock” Rosetta protocol [25, 46]). The complex architecture of Rosetta Broker enables also to efficiently combine de novo folding simulations with homology modeling, protein-protein or peptide docking and experimental data [24, 25]. Importantly, there is massive data concerning the Rosetta usage, settings and common problems encountered by users deposited in help files, FAQ or the Rosetta Commons Forum. Among very few other de novo methods for structure prediction of membrane proteins it is worth mentioning methods which are based on the identification of residue-residue contacts from multiple sequence alignments [47–49]. There are also fold-recognition methods for membrane proteins such as I-TASSER [50] or FILM3 [51] and a large number of homology-based methods often dedicated to only one protein family, e.g., G protein-coupled receptors (GPCR-I-TASSER [6], GPCRM [5], GOMoDo [8], GPCR-ModSim [7]) [52].

The accuracy of the generated hCNT3 model in its N-terminal part could be assessed, e.g., by scoring it against empirical data such as cross-linking data [53]. Unfortunately, no such data was available for the current study. Therefore, the validity of the N-terminal part and of the whole hCNT3 homotrimer model was assessed by molecular dynamics (MD). A 100 ns MD simulation was performed and heavy atoms RMSD with respect to the first frame was computed (see Figs. 3, 4, 5 and 6). The entire hCNT3 homotrimer structure and each monomer subunit (A, B and C) separately were stable during the whole 100 ns MD simulation with RMSD below 4 Å (see Fig. 3). Also, the three transmembrane helices which were predicted de novo by Broker were maintained in all three subunits during the MD simulation (see Fig. 4) though with slight deformations resulting in RMSD of the last simulation frame below 4 Å. The above results support the validity of de novo prediction of the N-terminal fragment of hCNT3.

Additionally, the stability of the selected helices during the MD simulation was examined in details (see Fig. 5). Two helices located in the binding site area: TMH7 and HP1 and one helix TMH9 located at the homotrimer interface were selected for the detailed analysis. The binding site residues were grouped in two sets after superposing our model on the vcCNT crystal structure which contained uridine and sodium ion inside the binding site. The first set, including Ile281, Thr280, Val249 and Asn246, Gly277, Gln251, Gly250, surrounded the sodium ion. The second set of residues consisted of Glu253, Thr252, Gln251, Glu429, Asn475, Ser478, Phe430, Phe473, Gly250, Phe278 and on TMH9: Leu356, Ile360, Asn359. That second set of residues surrounded the uridine ligand. Three residues: Thr280, Ile281 and Gly277 from the sodium ion binding residues set could be distinguished as quite stable during the MD simulation (RMSD values fluctuating around 2 Å). Residues from the uridine binding cluster were more flexible and were subjected to larger conformational changes (higher RMSD values around 3 Å were observed) (see Fig. 5, the right bottom panel). TMH9 located at the homotrimer interface was also flexible with RMSD reaching 4 Å (see the Fig. 5, the right top panel), as it was observed earlier [54]. Unfolding of HP1 in the absence of the sodium ion [54] was also observed (see Fig. 5, the left top panel), especially in the region between Val242 and Ser254. That corresponded to a significant change of RMSD from 2 to 3 Å around the frame no. 3200 (64 ns) for that region of HP1 (see Fig. 5, the right top panel). TMH7 was stable during the whole MD simulation with RMSD values fluctuating around 2 Å (see Fig. 5, the right top panel) as it was observed earlier [54].

In Fig. 6 SNPs for hCNT3 were displayed graphically. Most residues associated with non-synonymous amino acid changes (shown in red) were located in the extracellular region of the hCNT3 sequence which was quite flexible (RMSD values fluctuating around 4 Å – see the right bottom panel). Residues associated with synonymous changes (shown in yellow) were located in the middle part of the protein between lipid bilayers, yet a bit closer to the cytoplasmic side. Positions of those residues were stable during the simulation (RMSD values fluctuating around 2 Å – see Fig. 6, the right bottom panel)). In Fig. 6 one example SNP was shown in details (Gly277). Gly277 could be replaced with Arg. In such a case, a hydrogen bond could be formed with adjacent Gln251. That most probably could hamper binding of the ligand because Gln251 forms a hydrogen bond with uridine (see Fig. 2d). Biological studies confirmed the important phenotypic effect of that SNP. Namely, it was observed that mutation G↔A causing the mentioned above amino acid replacement Gly↔Arg resulted in the reduced uptake of inosine and thymidine in oocytes [39]. Another interesting example of non-synonymous SNP referred to Tyr23 located in the N-terminal part of TMH1 for which de novo structure prediction with Broker was performed. That SNP refers to the mutation A↔G which leads to the amino acid change Tyr↔Cys on the protein level. On the phenotype level that SNP has been related to ribavirin induced anemia, so most probably it causes the minor transporter activity, as it was suggested by Allegra et al. [55]. Referring back to the hCNT3 model presented in the current work, there was a close cysteine residue (Cys31) located in the bottom of TMH1 which could form a disulfide bridge with Cys23 replacing Tyr23. That could cause unfolding of that bottom part of TMH1. Another hypothesis explaining the structural effect of that SNP involved other residues: Phe248 (HP1), Phe163 (IH1) and Phe147 (TMH5) which were quite close to Tyr23. Interaction Cys-Phe is one of the strongest interactions in membrane proteins [56] so it is plausible that the amino acid replacement Tyr↔Cys could enable the new interaction Cys-Phe. What is more, Phe248 is located near the sodium ion, close to the middle part of HP1. As it was mentioned above in the description of the MD simulation, a noticeable movement of the middle region of HP1 away from the binding site towards TMH1 was observed in several low-energy frames recorded at the end of the simulation (see Fig. 5, the left top panel). It is plausible, that the amino acid change Tyr↔Cys enabling the mentioned above interaction Cys23-Phe248 could stabilize such a movement of HP1. That movement of HP1 away from the binding site might worsen the ligand - HP1 interactions and thus decrease the transporter activity. Nevertheless, more detailed studies regarding the impact of SNPs on the functioning of the hCNT3 transporter are certainly needed to confirm the above findings.

### CRFR1 receptor

**Fig. 7:**
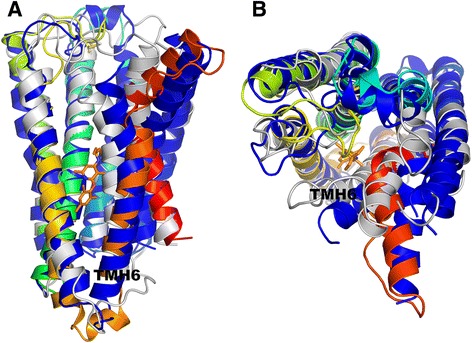
Comparison of three structures of CRFR1 receptor: a crystal structure (PDB id: 4K5Y) shown in dark blue with the antagonist CP-376395 shown in orange, a homology model of CRFR1 generated with MODELLER using the glucagon GPCR receptor template structure (PDB id: 4L6R) shown in grey, a Broker-refined model of CRFR1 shown in a blue-to-red color scheme. Here, the side view (**a**) and the top, extracellular view (**b**) of the receptor was shown

**Fig. 8:**
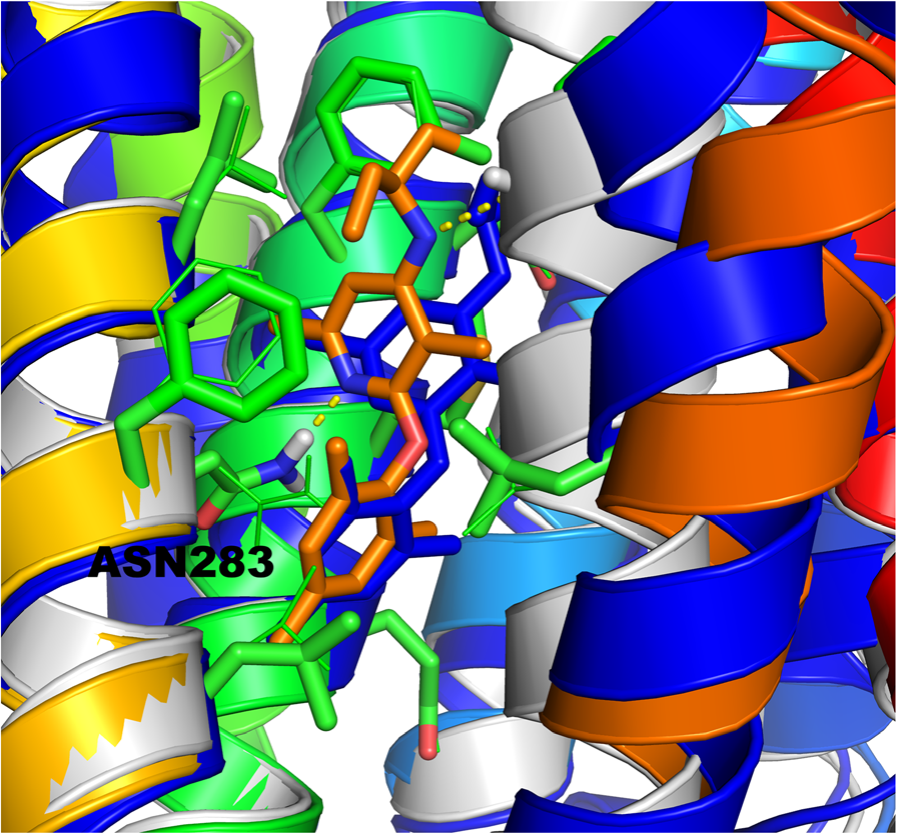
Comparison of the CP-376395 antagonist binding site of the CRFR1 crystal structure (dark blue), the homology model generated with MODELLER (grey), the homology model refined with Broker (blue-to-red color scheme with the ligand shown in orange). A few selected residues of the homology model refined with Broker were shown as green sticks. Those residues were selected to be flexible during the Autodock VINA docking. The starting side chain conformations of those residues (before docking) were shown with the wire representation while the resulting conformation (after docking) was shown with sticks. The polar contacts between the antagonist and the receptor were depicted with yellow dashed lines

Currently, the closest template for the CRFR1 receptor is the glucagon receptor GCGR (PDB id: 4L6R [36]). Although sequence identity is quite high (34%), the binding site of CRFR1 is located much deeper inside the receptor, than in the case of the GCGR binding site (see Fig. 7). Consequently, the binding site of CRFR1 with TMH6 moved away from the center of receptor is much more spacious than the GCGR binding site. For that reason, the typical homology modeling procedure would fail if the CRFR1 model was built using GCGR as a template. Indeed, the CRFR1 model generated with the typical homology modeling procedure (GPCRM) and the GCGR template structure provided the inaccurate CRFR1 model. The heavy atom RMSD with respect to the CRFR1 crystal structure (PDB id: 4K5Y) of the 8-residue fragment of TMH6 located inside the binding site was equal to 7.99 Å (see Table 1). The Broker large scale refinement of transmembrane helices in the CRFR1 model provided a more accurate conformation of TMH6 with RMSD equal to 3.41 Å (see Table 1). That large scale reconstruction of transmembrane helices in the CRFR1 homology model removed several atom clashes which were detected while docking the antagonist CP-376395 to that model (see Fig. 8 and Table 1). Those steric clashes were the main reason for the high repulsive energy of the ligand binding estimated with Autodock VINA (see Table 1). It is worth noting, that the Broker refinement of TMHs also made forming of the crucial polar contact between CP-376395 and Asn283 [28] possible. Here, the numbering of residues fits the PDB entry of CRFR1 (PDB id: 4K5Y). Additionally to the Broker large scale refinement of TMHs also several amino acid side chains surrounding the ligand (Leu287, Phe284, Leu280, Tyr327, Phe203, Asn202, Leu320, Glu209, Met206, Gln355, Leu323) were refined during the ligand docking. That approach further improved the quality of the CRFR1 binding site. The residues which were kept flexible during the ligand docking were depicted in the wire (before docking) and sticks (after docking) representation in Fig. 8.

**Table 1:** Autodock VINA affinity scores for the antagonist CP-376395 – CRFR1 complex

CRFR1 receptor structure	Heavy-atom RMSD of 8-residue fragment of 6TMH [Å]	Autodock VINA affinity score [kcal/mol]
Crystal	0.00	−9.46
Template-based	7.99	18.16
Broker-refined	3.41	−6.79

**Fig. 9:**
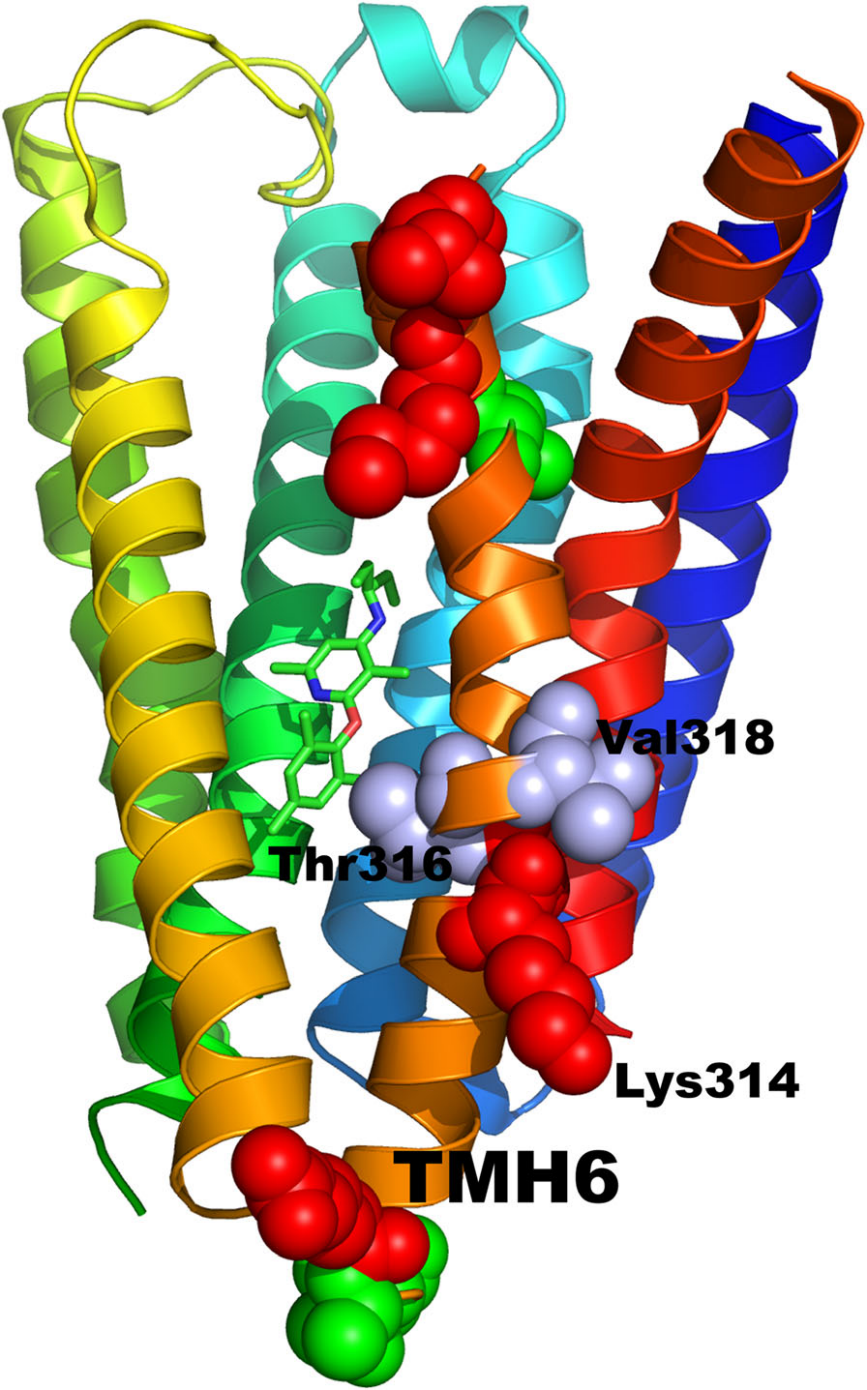
The CRFR1 crystal structure (PDB id: 4K5Y) with depicted the CP-376395 antagonist (green) and residues (shown as balls) to which non-synonymous mutations (red), synonymous mutations (green) and frame shift mutations (blue) refer

In Fig. 9 several residues in TMH6 were marked with the ball representation. They refer to SNPs reported in the literature for CRF-R1 (isoform 1 of the receptor) but were adjusted to the sequence of isoform 2 which was included in the PDB entry for CRFR1 (PDB id: 4K5Y). Here, other SNPs located in other TMHs were not shown for the sake of clarity. Residues associated with non-synonymous amino acid mutations (red) were located in the extra and intracellular part but also in the middle of TMH6. Residues associated with synonymous mutations were located only in the extra and intracellular part of TMH6. Two residues: Thr316 and Val318 associated with a mutation causing a frame shift (blue) were located in the middle of TMH6. The side chain of Thr316 was facing towards the CRFR1 binding site and was close to the ligand molecule. It was reported in the literature that it could be mutated to non-polar Ile316. Thus, the polar contact with the antagonist would be lost. Another residue, Val318 was facing the membrane, so most probably, if it was mutated to polar Cys a slight deformation of TMH6 in that region could be observed as a structural effect of that SNP. Polar Lys314 associated with non-synonymous amino acid mutation was located in the middle of TMH6 and was facing the membrane. It could be mutated to Arg or Asn. Most probably, such mutations could have an impact only on the TMH6 deformation induced by the change in the amino acid charge. To sum up, it could be hypothesized that mutations of Val318 and Lys314 could alter the bending of TMH6 and thus could change the size of the space accessible for the ligand binding. Thus, the strength of the ligand-receptor interactions could be changed. That could be relevant, for example, for the individual response to pharmacotherapy. Indeed, the described above SNPs are believed to be associated with the varied individual response to the treatment of asthma with inhaled corticosteroids (see the 225,965 entry in the ClinVar NCBI’s database).

## Conclusions

The current study supports that Rosetta Broker framework can be a versatile tool for de novo building and large scale reconstruction of transmembrane helices. It was able not only to reconstruct TMHs in such a way that kink angles were changed but also move away the whole TM helix away from the CRFR1 receptor center. Although no validation so far has been provided for the current de novo prediction of N-terminal TMHs in hCNT3, the MD simulation showed that such prediction was plausible.

It is certain that another detailed study regarding the hCNT3 transporter is needed to broaden the knowledge about its mechanism of action, the role of its single nucleotide polymorphisms and small molecules interactions. The current study is only one of the first steps in understanding the role of solute carriers transporters from the SLC28 family. The hCNT3 model built during this study could be used in future to manage sparse experimental data such as cross-links [53].

Building homology models for many of the receptors from the GPCR family is impeded by the lack of close homologs which could serve as templates. Here, one of the ways to work around that problem was shown. Namely, the large scale refinement of transmembrane helices in the CRFR1 homology model was shown to improve its overall accuracy and also its usefulness in ligand docking.

Theoretical models of hCNT3 and CRFR1 membrane proteins generated in this study were used for the analysis of SNPs. The role of SNPs in changing the protein structure or protein-ligand interactions was discussed and hypothetical structural changes caused by amino acid mutations were proposed. Nevertheless, biological studies should be performed to confirm those findings and to derive conclusions important for pharmacogenomics.

## Additional file

Additional file 1: Settings for the Rosetta Broker simulations. The Rosetta execution command included *minirosetta.mpi.linuxgccrelease* and flags presented in **Table S1**. Additional input files for the Rosetta Broker simulation are listed in **Table S2** together with their contents. (DOCX 15 kb)

## Abbreviations

CNT: Concentrative nucleoside transporter
CRFR1: Corticotropin-releasing factor receptor 1
GCGR: Glucagon receptor
GPCR: G protein-coupled receptor
MD: Molecular dynamics
SLCs: Solute carriers
SNP: Single nucleotide polymorphism
TMH: Transmembrane helix
