# Supplementary material for: Rosetta Broker for membrane protein structure prediction: concentrative nucleoside transporter 3 and corticotropin-releasing factor receptor 1 test cases
Source: BMC Struct Biol. 2017 Aug 3;17:8. doi: 10.1186/s12900-017-0078-8 (PMC5543540; doi:10.1186/s12900-017-0078-8)
Supplement: Additional file 1: — Settings for the Rosetta Broker simulations. The Rosetta execution command included minirosetta.mpi.linuxgccrelease and flags presented in Table S1. Additional input files for the Rosetta Broker simulation are listed in Table S2 together with their contents. (DOCX 15 kb) [file 12900_2017_78_MOESM1_ESM.docx]

**Supplementary material**

**Settings for the Rosetta Broker simulations.**

The Rosetta execution command included *minirosetta.mpi.linuxgccrelease* and flags presented in Table S1. Additional input files used for the Rosetta Broker simulation are listed in Table S2 together with their contents.

**Supplementary Table S1.** Flags for the Rosetta Broker simulations.

| -in:file:spanfile CNT3.span  -in:file:lipofile CNT3.lips4  -in:file:s CNT3.pdb  -run:protocol broker  -broker:setup setup_broker.tpb  -in:file:frag3 CNT3.frag.200.3mers  -in:file:frag9 CNT3.frag.200.9mers  -database $rosetta_path/rosetta_database/  -abinitio:stage2_patch score_membrane_s2.wts_patch  -abinitio:stage3a_patch score_membrane_s3a.wts_patch  -abinitio:stage3b_patch score_membrane_s3b.wts_patch  -abinitio:stage4_patch score_membrane_s4.wts_patch  -abinitio:membrane  -membrane:no_interpolate_Mpair  -membrane:Menv_penalties  -abinitio:close_loops  -abinitio:non_ideal_loop_closing  -score:find_neighbors_3dgrid  -out:file:fullatom true  -out:pdb true |
| --- |

**Supplementary Table S2.** Additional files used for the Broker simulation.

| **setup_broker.tpb**  CLAIMER SequenceClaimer  CMD_FLAG  END_CLAIMER  CLAIMER MembraneTopologyClaimer  END_CLAIMER  CLAIMER RigidChunkClaimer  # NO_USE_INPUT_POSE  PDB CNT3.pdb  REGION_FILE CNT3.rigid  END_CLAIMER |
| --- |
| **score_membrane_s2.wts_patch**  pair = 0.0  Mpair = 1.0  env = 0.0  Menv = 2.019  cbeta = 0.0  Mcbeta = 0.0  Menv_hbond = 2.019  Menv_termini = 2.019  Menv_tm_proj = 2.019  Mlipo = 1.0 |
| **score_membrane_s3a.wts_patch**  pair = 0.0  Mpair = 1.0  env = 0.0  Menv = 2.019  cbeta = 0.0  Mcbeta = 0.5  Menv_hbond = 2.019  Menv_termini = 2.019  Menv_tm_proj = 2.019  Mlipo = 1.0 |
| **score_membrane_s3b.wts_patch**  pair = 0.0  Mpair = 1.0  env = 0.0  Menv = 2.019  cbeta = 0.0  Mcbeta = 0.5  Menv_hbond = 2.019  Menv_termini = 2.019  Menv_tm_proj = 2.019  Mlipo = 1.0 |
| **score_membrane_s4.wts_patch**  pair = 0.0  Mpair = 1.0  env = 0.0  Menv = 2.019  cbeta = 0.0  Mcbeta = 2.5  Menv_hbond = 2.019  Menv_termini = 2.019  Menv_tm_proj = 2.019  Mlipo = 1.0 |
